# Rapid Reviews to Support Practice: A Guide for Professional Organization Practice Networks

**DOI:** 10.1177/00084174221123721

**Published:** 2022-10-13

**Authors:** Megan M. MacPherson, Rosalie H. Wang, Emma M. Smith, Gobika Sithamparanathan, Cara A. Sadiq, Anna RH Braunizer

**Keywords:** Evidence-based practice, Knowledge translation, Rapid review, Application des connaissances, pratique fondée sur des données probantes, revue rapide

## Abstract

**Background.** Occupational Therapists, among other healthcare decision makers, often need to make decisions within limited timeframes and cannot wait for the completion of large rigorous systematic reviews and meta-analyses. Rapid reviews are one method to increase the integration of research evidence into clinical decision making. Rapid reviews streamline the systematic review process to allow for the timely synthesis of evidence; however, there does not exist a single agreed upon guide for the methodology and reporting of rapid reviews. **Purpose.** This paper proposes a rapid review methodology that is customized to a professional organization practice which can feasibly be used by practice networks such as those of the Canadian Association for Occupational Therapy to conduct reviews. **Implications.** Practice networks provide a sustainable mechanism to integrate research evidence and foster communication amongst practitioners. This guide for conducting and reporting rapid reviews can be used across Occupational Therapy practice networks and similar groups to support the consistent and timely synthesis of evidence necessary to improve evidence-informed clinical decision making.

## Résumé

**Description.** Les ergothérapeutes, comme de nombreux professionnels de la santé, doivent souvent prendre des décisions dans des délais limités, sans attendre la réalisation de grandes revues systématiques rigoureuses et de méta-analyses. Les revues rapides sont une méthode permettant d’intégrer davantage les données de recherche dans la prise de décisions cliniques. Elles consistent à rationaliser le processus de revue systématique pour permettre la synthèse des données en temps opportun; cependant, il n’existe pas de guide faisant l’unanimité pour la méthodologie et la reddition de comptes relatives aux revues rapides. **But.** Cet article propose une méthodologie de revue rapide adaptée à la pratique d’une organisation professionnelle et pouvant être utilisée par des réseaux de pratique comme ceux de l’Association canadienne des ergothérapeutes (ACE). **Implications.** Les réseaux de pratique offrent un mécanisme viable pour intégrer les données de recherche et favoriser la communication entre praticiens. Ce guide pour réaliser des revues rapides et en rendre compte peut être utilisé dans les réseaux de pratique en ergothérapie et dans d’autres groupes du même type pour favoriser une synthèse opportune et cohérente des données nécessaires pour améliorer la prise de décisions cliniques basée sur des données probantes.

## Introduction

Research evidence can take up to 17 years to be implemented into clinical practice (Morris et al., 2011). This delay in knowledge translation is prohibitive for Occupational Therapists who aim to integrate research evidence into their clinical decision making (Bennett & Bennett, 2000). To build capacity and improve the translation of research knowledge into Occupational Therapy policies and practice, it is important to understand what barriers are impeding the uptake of research evidence into clinical decision making. It has been consistently cited that lack of timely and relevant research outputs, coupled with inadequate access to relevant sources are among the largest barriers to integrating research into practice (Oliver et al., 2014; World Health Organisation, 2012). One way to improve the accessibility of research evidence and expedite its translation to clinical practice is through rapid reviews of the literature. Evidence syntheses, such as rapid reviews, have been suggested as an effective knowledge product for evidence-based decision making (Chambers et al., 2011; Grimshaw et al., 2012).

Occupational therapy practice areas that are developing very quickly and that require rapid response (e.g., technological interventions, COVID-19 pandemic response, and transition to telerehabilitation services) can be overwhelming for clinicians and decision makers (Dahl-Popolizio et al., 2020; Mattison et al., 2020; Wang et al., 2022). Professional practice networks provide a forum in which professionals can connect, communicate, and collaborate with others who have similar interests. Often these networks have a goal to promote excellence in practice and develop new knowledge or resources to improve practice (e.g., https://ppno.ca/; https://caot.ca/site/pd/otn?nav = sidebar). While reviews of the literature are often conducted within an academic setting, professional organization practice networks can play a leadership role in conducting relevant reviews that meet clinician needs and provide actionable evidence in an efficient manner. If a rapid review is conducted by and for a specific practice network, it is more likely to result in actionable evidence which will be integrated into practice while also building capacity of professionals through the conduct of the review itself. Using rapid reviews, the Canadian Association for Occupational Therapists Technology for Occupation and Participation (CAOT TOP) practice network aims to provide timely information regarding the rapidly growing field of technology in practice to provide relevant information for practicing Occupational Therapists.

### What is a Rapid Review?

The Canadian health and social care systems face increasingly complex challenges that require both the generation and amalgamation of knowledge in short periods of time. For example, policymakers often require evidence to support time-sensitive decision-making pertaining to the efficiency, quality, and equity of programs and services. Systematic reviews provide a synthesis of existing evidence for a specific topic and are being increasingly employed in policymaking and practice (Bosch-Capblanch et al., 2012; Oxman et al., 2010); however, the time and cost intensive nature of systematic reviews poses a barrier in supporting time-efficient decision making (Oliver et al., 2014).

Rapid reviews are one alternative to the cumbersome systematic review process which provide relevant evidence in a time- and cost-efficient manner (Tricco et al., 2015). Specifically, rapid reviews are a form of knowledge synthesis in which the steps taken in a systematic review are streamlined to produce actionable evidence in a shorter time (Polisena et al., 2015) (see Table 1 for key methodological differences between rapid and systematic reviews). Rapid reviews have been increasingly used in health systems policy-making, health-related intervention development, and health technology assessment (Harker & Kleijnen, 2012; Polisena et al., 2015; Watt et al., 2008).

**Table 1:** Key Methodological Differences Between Rapid and Systematic Reviews

	Rapid review^a,b^	Systematic review^c^
**Definition**	Time-efficient synthesis of the literature which streamlines traditional review processes	Comprehensive and rigorous synthesis of the literature
**End-user engagement**	High	Low
**Timeframe**	1–6 months	>1 year
**Searches**	Often apply limits to year and language of publication	Comprehensive
**Synthesis**	Descriptive summary of findings	Descriptive summary which may include meta-analysis
**Dissemination**	Dependent on end-user needs	Peer review journals, academic conferences

^a^ Featherstone et al. (2015).

^b^ Hartling et al. (2015a)

^c^ Higgins et al. (2019).

While rapid reviews provide a synthesis of information in a timely manner, they are not without their faults. In order to improve the timeliness of the review process, rapid reviews often make sacrifices on the rigour included in the review resulting in a less accurate and robust review in comparison to a systematic review (Featherstone et al., 2015). Further, without a standardized methodology in place, as is seen in other review types such as systematic reviews (Higgins et al., 2019), it can be difficult to determine how to best conduct and report a rapid review.

Based on previous reviews of the literature, numerous approaches to conducting rapid reviews have been identified (Haby et al., 2016; Hamel et al., 2021; Tricco et al., 2015). While other researchers and groups have proposed approaches for rapid reviews (Abrami et al., 2010; Featherstone et al., 2015; Khangura et al., 2012; Thomas et al., 2013; Tricco et al., 2016; Varker et al., 2015), there is no agreed upon methodology and these approaches, and they were primarily developed for the purpose of rapid reviews being conducted by individual researchers. This guide consolidates and builds on previous approaches and proposes a rapid review methodology which is tailored for professional organization practice and can be sustainably implemented within the CAOT TOP practice network.

### The CAOT Practice Network: Technology for Occupation and Participation

As technology use continues to rapidly advance, engaging in evidence-based practice through the integration of research evidence into clinical decision making is particularly challenging among Occupational Therapists intending to use technologies in their practice to improve clients’ occupational performance and engagement. As technology applications continue to expand and become increasingly integrated into daily occupations, Occupational Therapists’ knowledge and skills within technology practice must also continually grow (Mattison et al., 2020; Wang et al., 2022).

Within the CAOT, the practice network: *Technology for Occupation and Participation (TOP)* aims to build capacity and take action in developing and implementing policies and practices involving technology by supporting research related to evaluating Occupational Therapy and participation outcomes with technology-oriented interventions (https://caot.ca/site/pd/networks/technology?nav = sidebar). The network is comprised of volunteers who typically have full time jobs and students with limited time, but who are eager to advance practice. As such, we included in our proposed methodologies constraints that can help expedite the review process in a manner that can ensure for example, the completion of one rapid review per year by the CAOT TOP Practice Network.

Motivated by the purpose of strengthening Occupational Therapy practice relating to the development and provision of technology, we developed this guide to provide suggestions on how to plan, conduct, and disseminate rapid reviews in the context of professional organization practice networks. It is important to note that there is no “one-size-fits-all” approach for rapid reviews and all suggestions within this guide are just that, suggestions for what may be included to streamline the review process. Constraints placed on a review should be dependent on time and available resources while still upholding rigour in the review process (Tricco et al., 2022).

## Rapid Review Process: An Overview

**Figure 1.**
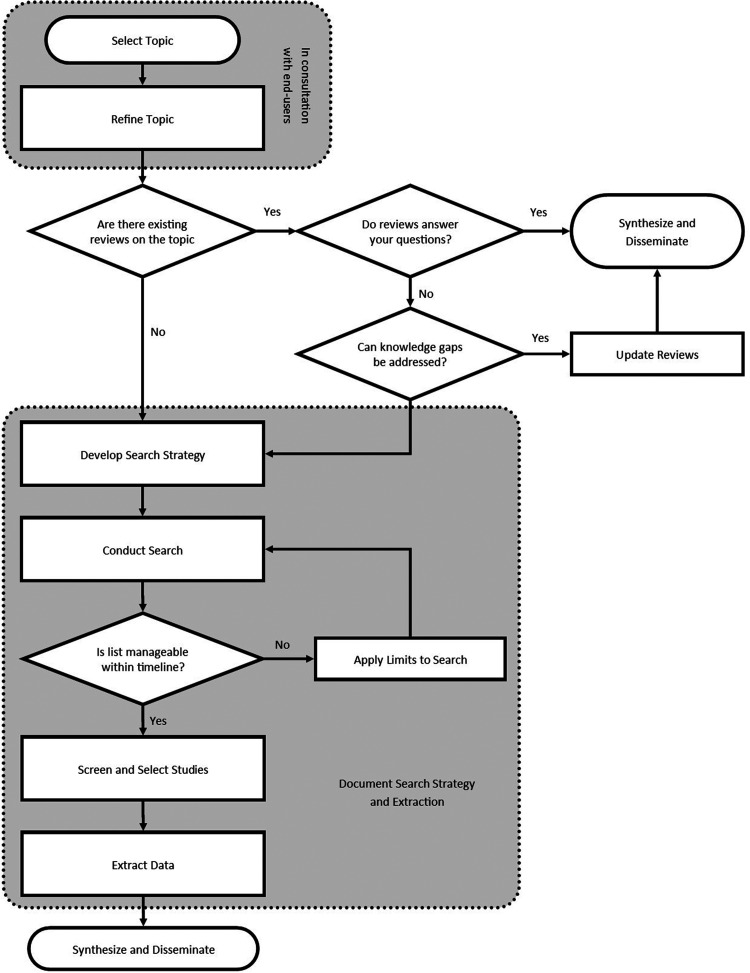
Rapid Review Process Map.

Early and continued engagement with end-users is essential for rapid reviews to ensure that the limitations employed throughout allow for actionable and relevant information for Occupational Therapists. Rapid reviews draw from systematic review methods and the same broad stages are followed; however, rapid reviews can be streamlined at all review stages from the research question to disseminating results. In order to maintain methodological rigour and transparent reporting, decisions and rationale for all limits placed on a rapid review should be thoroughly documented (Plüddemann et al., 2018). See Table 2 for an overview of review stages and the approaches that can be taken to streamline those stages within a rapid review. Based on previous recommendations on how to conduct rapid reviews, we piloted a rapid review within the CAOT TOP Practice Network (MacPherson et al., 2022). Our decisions, and how this process will be implemented within the CAOT TOP practice network, will be described in the following steps where relevant. See Figure 1 for a process map outlining these steps.

**Table 2:** Overview of Rapid Review Approaches

Review stage	Rapid review approaches	Potential bias introduced
**1. Topic selection, and refinement**	Those requesting a specific review should be included in topic refinement so the purpose of the review can be clearly defined, and assurances can be made from the onset so that the resulting review answers key questions of importance to Occupational Therapy practice.	
**2. Develop search strategy**	To streamline the search, many rapid reviews place limits on date, language, and study design, geographical location. Many rapid reviews use a staged search approach in which they first limit the search to existing reviews (if they can answer the research question by summarizing existing reviews the search stops here), then studies with other designs that provide the most rigorous evidence to answer the research question.	Excluding grey literature will invariably exclude unpublished data and negative results. Excluding articles based on year of publication, language or geographic location may not capture the full extent of the literature and exclude potentially significant and relevant studies.
**3. Screening and study selection**	Common limits for rapid reviews include study screening and selection completed by a single reviewer.	Less transparency and reproducibility.
**4. Data extraction & Risk of bias assessment**	Common limits include data extraction and risk of bias (where appropriate) completed by a single reviewer. Data extraction is often limited to only key study characteristics and outcomes needed to answer the key questions identified in stage 1.	Increased errors.
**5. Evidence synthesis & Dissemination**	Rapid reviews commonly result in a narrative summary of the literature. Results often include implications for occupational therapists, policy or healthcare delivery recommendations, and limitations of the research. Rapid reviews can be reported in the way that best meets end-user needs (peer review journals, internal reports, rapid report briefs, social media, etc.)	

### Stage 1: Topic Selection and Refinement

Rapid reviews should be conducted using a person-centered and participatory action research approach including Occupational Therapy scholars, clinicians, and other community members (e.g., patients, other health professionals, policy makers) to collaboratively identify and address health or social challenges faced by the community (Asaba & Suarez-Balcazar, 2018; Minkler & Wallerstein, 2011; Satcher, 2005). Professional organization practice networks are well poised to bridge the gap between scholars and clinicians, allowing for the co-creation of knowledge through rapid reviews towards developing applicable solutions to community identified topics. By linking both theoretical and empirical knowledge with real-world issues within Occupational Therapy practice, all individuals within the CAOT TOP practice network (i.e., scholars and clinicians) can collaborate to impact both occupational science and Occupational Therapy practice (Asaba & Suarez-Balcazar, 2018).

Within the CAOT TOP network, rapid reviews will only be conducted on topics nominated by an end-user (i.e., a member of the network or other Occupational Therapist or relevant community member) and that have been agreed upon by the network as a high value area for a rapid review. For example, topic ideas may be generated within TOP Practice Network knowledge sharing circles and via strategic planning surveys.

Early and continued engagement with end-users allows researchers to narrow the scope of a review while ensuring that the question being answered is useful and relevant to knowledge users (Garritty et al., 2020). This is of the utmost importance, because in rapid reviews, there are often trade-offs dependent on end-user timelines. To complete a review in a timely fashion, researchers should consult with end-users to narrow the scope of the review topic. Limiting the number of questions, interventions, and outcomes to be targeted in a review can decrease the time needed to answer those question. In order to narrow the scope, it is imperative to understand the end-user needs, their intended use of the review, and their timeframe (Hartling et al., 2015a; Khangura et al., 2012; Thomas et al., 2013). Within the CAOT TOP network, a brief abstract (3–5 sentences) will be posted internally to notify all network members of ongoing and upcoming reviews and allow for iteration on the research question based on additional end-user needs. Other organizations are encouraged to do the same to allow for the resulting review to have the greatest impact on policy and practice by being tailored to a variety of end-user needs.

### Stage 2: Develop a Search Strategy

All rapid reviews should make available the full search strategy for at least one database they have searched (Moher et al., 2010). Two or more relevant databases are often searched for rapid reviews with the following databases most commonly cited: PubMed, Embase, and the Cochrane Library (Abou-Setta et al., 2016; Polisena et al., 2015; Tricco et al., 2015). Additional databases that are commonly used within Occupational Therapy reviews and should be considered include CINAHL, Web of Science, and PsycInfo (e.g., Bauer et al., 2022; Marcotte et al., 2020; Wallis et al., 2020). Further, involving subject matter experts and healthcare librarians with experiences in reviews can aid in the efficient development of a comprehensive search strategy (Dudden & Protzko, 2011; Lefebvre et al., 2019).

Many rapid reviews streamline the systematic review process by limiting the dates, languages, geographical area, or study design within the search strategy (Abou-Setta et al., 2016; Hartling et al., 2015b; Polisena et al., 2015; Tricco et al., 2015). Grey literature (i.e., non-peer reviewed reports) searches may be essential for certain topics, but are another common way researchers can limit the search strategy if necessary. Based on the research experiences within the CAOT TOP, the following potentially relevant grey literature resources within the context of Canadian Occupational Therapy were identified: clinicaltrials.gov, theses and dissertations, professional associations’ conference proceedings, and the McMaster Health Forum (https://www.healthsystemsevidence.org/; https://www.socialsystemsevidence.org/).

Similar to Garritty et al., (2020), we recommend using a staged approach where appropriate, in which researchers start by identifying existing reviews of the literature. If a synthesis of existing reviews can be used to provide a robust answer to the research questions, the search stops here. If there are still gaps the researcher and end-user feel are necessary to answer in this review, then the review can be updated by identifying recent studies that have been published since the most recent included review. Researchers can limit this additional search, if necessary, to only those study designs that provide the most rigorous evidence to answer their questions. If there are no current reviews of the literature, review authors can look at individual studies (but may limit to the study design that can most appropriately answer their question).

### Stage 3: Screening and Study Selection

As previously mentioned, it is important for a robust search to include multiple databases; however, this will likely result in overlap between the various databases. Within the screening step, management of data overlap through de-duplication must be conducted to prevent reviewers from screening one source multiple times (McKeown & Mir, 2021). Reviewers should consider using specialized software such as Covidence or reference managers such as EndNote, Zotero, or Mendeley to expedite the review process by automatically de-duplicating search results. Within a recent evaluation of the performance of different methods for de-duplicating references, McKeown and Mir (2021) noted that Covidence was among the most accurate and efficient methods for de-duplication, and it significantly outperformed reference management software. When choosing a software program to aid in the review process, McKeown and Mir (2021) note that reviewers should consider not only the de-duplication performance of a software, but also the availability of other program functionalities to aid in screening references, resolving conflicts, and data extraction. Covidence is one of the more comprehensive tools which can aid in all review steps, as such we recommend it to streamline the review process and reduce the need for the review team to use multiple software and programs to complete their rapid review.

Following de-duplication, reviewers can screen each title and abstract then full text for eligibility. Whereas systematic reviews typically require two independent coders at the screening phase, rapid reviews can expedite the time to complete a review by limiting these phases to a single coder (Abou-Setta et al., 2016; Hartling et al., 2015b; Polisena et al., 2015; Tricco et al., 2015). It is important to note that, while use of a single reviewer has been estimated to reduce screening time by 60%, it also results in 8%–20% of eligible studies being missed (Edwards et al., 2002; Glasziou et al., 2002; Shemilt et al., 2016). Given this level of error, it is recommended that dual reviewers be used (when possible) in the screening phase to ensure the accuracy of the pool of articles to be synthesized. When this is not possible, we recommend that two reviewers dual screen approximately 25% of the studies (Garritty et al., 2020). Additionally, we recommend that authors include an audit trail (Carcary, 2009). Typically used in qualitative research, an audit trail can be applied to rapid reviews to improve transparency and trustworthiness of the research by providing a record of how the review was carried out and how decisions were arrived at by the reviewers (Carcary, 2009). By making available an audit trail, authors are challenged to be intentional with their record keeping and decision-making processes throughout the review.

When beginning title and abstract screening, we recommend that a standardized screening form be developed which thoroughly defines the inclusion and exclusion criteria (Tricco et al., 2017). Eligibility criteria should be well-defined, use clear and unambiguous language, and thorough explanation and elaboration should be provided to reviewers to define concepts and terms. This explanation and elaboration document can support the reviewers with study selection and ensure that eligibility criteria are being applied consistently. Additionally, the inclusion of content experts who have experience conducting reviews can be useful for an expedited screening process (Lefebvre et al., 2019). The screening form, along with the explanation and elaboration document, should be piloted and refined by the review team on 5 to 10 articles (Garritty et al., 2020) and an audit log should be used to document key decisions made (Tricco et al., 2017). By piloting these documents, the review team can identify potential discrepancies in how an eligibility criterion is being applied, and provide additional examples and definitions where necessary.

Eligibility criteria should be based on the research question; however, some limits commonly placed within rapid reviews to limit the potential pool of eligible resources are as follows: date of publication, geographical location, language, age range, and study design (Garritty et al., 2020). If there are justifiable reasons to limit your search (e.g., you are reviewing apps so limit the date range to when the app stores were first introduced, no review team members speaking another language, etc.), include these reasons within your audit log and place those constraints at the onset. Once these justifiable limits are placed on your search strategy, we recommend running a search without placing any additional arbitrary limits, and only applying additional limits if the pool of potential studies is too large for your given timeline – this can help to ensure that you are retrieving as many potentially relevant sources as possible prior to introducing bias with these arbitrary limits (Tricco et al., 2017).

Previous work has described the screening phase as one of the most difficult and time consuming among systematic reviews (Carver et al., 2013). To improve the flow within the screening process and reduce clerical errors required to keep track of all potentially relevant articles, we recommend that those wanting to conduct a rapid review use abstract screening software such as Covidence and complete a PRISMA flow diagram to document the article search and selection process. If the review team does not have the resources available to purchase specialized software, excel may also be used within the screening and data extraction steps of the review; however, use of specialized software such as Covidence can aid in streamlining the review process (McKeown & Mir, 2021). Covidence allows multiple reviewers to assess the eligibility of each source at the same time, select pre-specified reasons when excluding an article, tracks eligibility decisions as they are made, and remove an article from the review queue in real time (Kellermeyer et al., 2018). If two reviewers are included within the screening stage, Covidence has a conflict resolution workflow integrated into its design to allow for all sources in which there was a conflict to be easily located and resolved (Kellermeyer et al., 2018). Further, Covidence produces a PRISMA flow diagram, thereby reducing the burden on researchers to manage the number of studies screened and reasons for exclusion.

### Stage 4: Data Extraction & Risk of Bias Assessment

#### Data extraction

This stage in the rapid review process includes extraction of all relevant data from those resources identified as eligible during the screening stage. We at the CAOT TOP Practice Network will be working in collaboration with members of the network as well as Occupational Therapy students during the extraction stage. Those involved in creating the research question will collaboratively develop a customized data extraction tool which will help other reviewers extract relevant data and critically appraise the literature. The efficient translation of research evidence via rapid reviews into health systems requires community knowledge to ensure that data being extracted is of interest to them (Jull et al., 2017). Further, by working with those who are interested in the practice area, and including them in the data extraction process, we hope to build their capacity while also ensuring that the data collected in the review is relevant to those who will be using the review outcomes in practice.

To streamline the data extraction, reviewers should work with end-users to identify what information is necessary to extract and the data extraction should be limited to a minimal set of required items (Tricco et al., 2017). Having engaged with Occupational Therapists, decision-makers, content experts, patients, and caregivers at the outset to develop specific research questions, we can prioritize which outcomes are most important and relevant to practice and ensure that all relevant information is collected and synthesized. Use of data extraction forms which provide explanation and elaboration on relevant items can ensure consistency in extraction (Garritty et al., 2020).

Within the CAOT TOP Practice Network, when data extraction includes a novice reviewer, a network lead or expert reviewer will complete data extraction for a random 20% of studies. This data will be compared to that extracted by novice reviewers to ensure the accuracy of data collection. We recommend a single extractor with verification, as past research has estimated that, compared to dual extraction, single extraction with verification resulted 36% less time and 22% more errors; however, these errors rarely cause changes to a reviews results or conclusion (Buscemi et al., 2006).

#### Risk of bias assessment

Critical appraisal of the quality of the research being synthesized and quality of methods employed for each included resource is standard within systematic reviews (Higgins et al., 2019); however, this step is conducted selectively within rapid reviews based on the aim of the review (Polisena et al., 2015). For example, when the purpose of a review is to scope the literature, not to evaluate specific effects, a risk of bias assessment may not be necessary (Tricco et al., 2017). When conducting a rapid review of reviews, researchers may wish to accept the summary assessment of risk of bias conducted within existing reviews; however, they may wish to examine the risk of bias within the reviews themselves. Like data extraction, we recommend use of a single reviewer with verification by another reviewer when this step is necessary (Garritty et al., 2020). Different risk of bias and quality assessment tools are available and are often chosen based on specific study designs being assessed. Below we provide a list of commonly used risk of bias tools (note this is not a comprehensive list): A Risk of Bias Assessment Tool for Systematic Reviews (ROBIS) (Whiting et al., 2016); Physiotherapy Evidence Database (PEDro) (Maher et al., 2003), and Risk of Bias 2 (RoB 2) (Sterne et al., 2019) for RCTs; Risk Of Bias In Non-randomised Studies-of Interventions tool (ROBINS-I) (Sterne et al., 2016); and Appraisal of Guidelines for Research & Evaluation Global Rating Scale (AGREE GRS) (Brouwers et al., 2012) to appraise clinical practice guides. Additionally, The Critical Appraisals Skills Programme (CASP) (https://casp-uk.net/casp-tools-checklists/), Scottish Intercollegiate Guidelines Network (SIGN) (https://www.sign.ac .uk/what-we-do/methodology/checklists/), and Joanna Briggs Institute (JBI) (https://jbi.global/critical-appraisal-tools) provide a number of tools for different study designs which can be found at their respective websites.

### Stage 5: Evidence Synthesis & Dissemination

Results from research syntheses have the potential to play a major role within the healthcare system (Lavis et al., 2005). Whereas systematic reviews often conduct meta-analyses to answer causal questions, rapid reviews often provide a narrative synthesis of the included resources (Garritty et al., 2020; Tricco et al., 2017). Evidence synthesis and dissemination of rapid review outcomes should be tailored for use within specific contexts to ensure that research evidence can be effectively and efficiently implemented into clinical practice (Graham et al., 2006; Graham & Tetroe, 2010). How the evidence in a rapid review is synthesized depends on end-user needs and can include conclusions, recommendations, implications for policy, a table or repository of tools, etc. Experts recommend that all rapid reviews clearly state what steps were taken to streamline the process and discuss potential limitations arising from those methodological decisions (Abou-Setta et al., 2016; Hartling et al., 2015b; Watt et al., 2008). Examples of some potential bias introduced based on methodological choices can be seen in Table 2.

Dissemination of results involves communicating the results of the review to a specific audience with the goal of maximizing uptake and impact (Straus et al., 2013). As the purpose of rapid reviews is most often to help inform clinical decision making and policy decisions, the ways in which they are disseminated should be customized for each review by considering the target audience and the anticipated impact of the review on practice (Tricco et al., 2017). This may include posting results on an organizational website, presenting at stakeholder meetings or workshops, rapid response briefs (a summary of the best available evidence presented in direct response to the initial topic question) published in Occupational Therapy practice magazines, publishing full reports in peer-reviewed journals, creating online databases or repositories, or sharing results via social media or email distribution to key knowledge users. Where important topics exist that are likely to evolve over time, we recommend having “living” rapid reviews. Similar to living systematic review, living rapid reviews can provide up-to-date summaries which are updated as new information becomes available (Elliott et al., 2014; Kelly et al., 2022). For example, the research regarding mHealth apps is likely to continually grow. If wishing to develop a repository of apps for clinicians to recommend to clients, having a living review in which results are updated on an annual or semi-annual basis is warranted. Although producers of rapid reviews have access to the same dissemination tools and distribution channels as systematic reviews, they should prioritize the practical needs of the knowledge user over traditional or academic approaches to dissemination (Tricco et al., 2017).

While not all knowledge products will require in-depth discussion of the methods used, we recommend having available, at end-user request, a rapid review report and audit log detailing the methodologies, strengths and weaknesses of the review process used, and results which lead to the conclusions within the final knowledge product presented to end-users (to improve transparency, these documents can be posted on online repositories such as open science framework https://osf.io/). We also recommend that within all knowledge products, it is acknowledged that these results were garnered from a rapid review and the potential limitations this has on the impact of the review findings (Tricco et al., 2017). See Table 3 for a list of recommendations made within this guide.

**Table 3:** Key Recommendations for Conducting Rigorous Rapid Reviews

Recommendations	Explanation
Make transparent methodological choices to expedite the review process.	These choices should be informed by stakeholder needs and must ensure that the review can appropriately answer key stakeholder driven questions.
Use software to automate and track review steps.	Use of computer software such as endnote and Covidence can enhance transparency and timeliness of a review by generating values for use in a PRISMA flow chart. Using these software can assist researchers by making various steps in the rapid review process more efficient.
Engage with end-users early and often.	End-users should be the ones driving the research question, eligibility criteria, data extraction, and dissemination. It is integral to collaborate with end-users in decision making throughout the review process to ensure that the review meets end-user needs and to improve uptake and impact of the review once completed.
Create explanation and elaboration documents to ensure consistency and transparency.	Explanation and elaboration documents should be developed to define key terms, provide examples, and elaborate of specific eligibility criteria or items to be extracted. If the reviewer has a thorough understanding of what and why certain information is needed (and has written guidance for when they are unsure), they are more likely to be able to consistently apply these criteria during the screening and extraction phases. Having these documents can also aid in the rapid review report by providing a transparent record of what and why certain eligibility criteria and data extraction items were included.
Time permitting, use two reviewers for screening stage.	Given time and resources, you will have a more robust and rigorous review if all titles and abstracts, then full text screening is conducted independently by two reviewers. If there is insufficient time, have screening completed by one reviewer and have ∼25% double screened to ensure consistency.
Time permitting, have 20% of data extraction and risk of bias assessment verified by an expert coder.	Having this additional check within data extraction can ensure that results are accurate and that the novice reviewer has consistently applied the data extraction form to all included resources.
Use experts throughout to streamline the process.	Subject matter librarians can aid in developing the search strategy, review experts can aid in running the search and using review software, content experts can aid in verification of screening and data extraction if necessary.
Create a rapid review report outlining key methodological choices and limitations and provide a disclosure of limitations within all knowledge products.	While not all end-users will value or need the full rapid review report, it is necessary for transparency and can aid end-users in decision making by providing insights into the methods used, the quality of the review and included studies, and limitations. By understanding the limitations within the methodological choices, this can aid decision makers in knowing the scope of the review and how far the results can be extended (i.e., can they make causal claims from your rapid review or do they need to exercise caution when using these results to imply a causal relationship between an assistive device and clinical outcome?)

## Conclusion

Occupational Therapists, like other healthcare decision makers, require the timely synthesis of research evidence to improve their ability to integrate research evidence into their practice; however, traditional systematic reviews are too time and resource intensive. For example, within the field of health-related technology, technological advancement is likely to outpace research resulting in limited utility of systematic reviews on outdated technology. Rapid reviews are an efficient way to translate valuable research evidence into policy and practice decision making; however, no standard methods exist for the conduct and dissemination of rapid reviews tailored to professional practice networks and Occupational Therapy practice. Practice Networks and professional groups provide a sustainable mechanism in which rapid reviews can be conducted and efficiently communicated to end-users. This paper provides a guide to rapid reviews which will continue to be used within the CAOT TOP practice network and can be applied more broadly for Occupational Therapy rapid reviews and used by other professional organizations. It is hoped that the recommendations presented in this article are helpful for the conduct of rapid reviews and professional organization practice, can help build capacity, and aid in the timely translation of relevant research evidence into clinical practice.

## Key Messages

- Rapid reviews, when conducted in a rigorous manner with transparent reporting, can be integral to the integration of research evidence into clinical decision making.
- This work synthesizes previous rapid review guides and proposes rapid review mechanisms relevant to professional organization practice. Use of this guide within the CAOT TOP network will help to build capacity and take action in implementing research-based technology into Occupational Therapy practice.
- It is anticipated that the rapid review guide presented may be easily adapted to other professional organizations and practice networks to improve the timely translation of knowledge to practice.
